# Social Media Release Increases Dissemination of Original Articles in the Clinical Pain Sciences

**DOI:** 10.1371/journal.pone.0068914

**Published:** 2013-07-17

**Authors:** Heidi G. Allen, Tasha R. Stanton, Flavia Di Pietro, G. Lorimer Moseley

**Affiliations:** 1 Sansom Institute for Health Research, University of South Australia, Adelaide, Australia; 2 Neuroscience Research Australia, Sydney, Australia; 3 University of New South Wales, Sydney, Australia; Children’s Hospital of Eastern Ontario, Canada

## Abstract

A barrier to dissemination of research is that it depends on the end-user searching for or ‘pulling’ relevant knowledge from the literature base. Social media instead ‘pushes’ relevant knowledge straight to the end-user, via blogs and sites such as Facebook and Twitter. That social media is very effective at improving dissemination seems well accepted, but, remarkably, there is no evidence to support this claim. We aimed to quantify the impact of social media release on views and downloads of articles in the clinical pain sciences. Sixteen PLOS ONE articles were blogged and released via Facebook, Twitter, LinkedIn and ResearchBlogging.org on one of two randomly selected dates. The other date served as a control. The primary outcomes were the rate of HTML views and PDF downloads of the article, over a seven-day period. The critical result was an increase in both outcome variables in the week after the blog post and social media release. The mean ± SD rate of HTML views in the week after the social media release was 18±18 per day, whereas the rate during the other three weeks was no more than 6±3 per day. The mean ± SD rate of PDF downloads in the week after the social media release was 4±4 per day, whereas the rate during the other three weeks was less than 1±1 per day (p<0.05 for all comparisons). However, none of the recognized measures of social media reach, engagement or virality related to either outcome variable, nor to citation count one year later (p>0.3 for all). We conclude that social media release of a research article in the clinical pain sciences increases the number of people who view or download that article, but conventional social media metrics are unrelated to the effect.

## Introduction

The impact of research is fundamentally dependent on how well it is disseminated to the end-user. Conventional routes of dissemination involve journal publications, conference presentations and, ultimately although often years later, textbooks. This model of dissemination requires the end-user to search for, or ‘pull’, the relevant knowledge from the literature base [1]. With regard to health and medical research, this approach might be ineffective because the end-users are often clinicians who do not subscribe to journals, nor attend conferences. The rise of open access publication reduces one barrier to effective dissemination by making literature freely available for all who wish to consult it, but it still relies on the end-user pulling out the relevant knowledge [2]–[3].

The rapid rise in popularity of web logs (blogs) and social media sites such as Facebook and Twitter, has positioned them as critical tools with which to aid dissemination. Health and medical research is no exception - high profile journals such as the New England Journal of Medicine (NEJM) and the British Medical Journal (BMJ) have established cohesive digital strategies that incorporate both blogs and social media sites (Table S1), presumably in the hope of improving the dissemination of knowledge. This approach contrasts with the pull approach insofar as it ‘pushes’ the knowledge to the end-user [1]. By having different blog and social media sites, journals allow the end-user to self-select the genre of knowledge they wish to receive. RSS (Really Simple Syndication) is another example of how users can self-select information. Although not a pure social media tool, RSS feeds enable the pushing of individualised information and blog contents. RSS permits some user interaction and information sharing.

**Figure 1:**
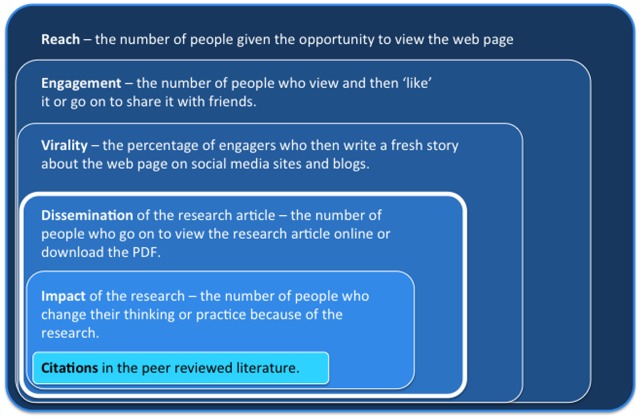
Terms and definitions. Key concepts concerning social media metrics and what is arguably true research impact – achieving shifting practice or thinking, as distinct from the conventional although controversial measure of research impact - citations. We took dissemination of the research article, as measured by the number of unique users who viewed the HTML or downloaded the PDF of the article, as the most proximal estimate of true impact that we could measure. As impact is likely to reflect a proportion of dissemination, so too does dissemination reflect a small proportion of the social media metrics commonly used to reflect impact. The results of our study show clearly that although social medial release increases dissemination, the social media metrics do not relate to dissemination, nor to citation count a year later.

The fundamental importance of a digital strategy is emphatically stressed by social media advocates [4]. Markers such as the number of ‘likes’, or the number of Facebook or Twitter followers are cited as measures of research impact, collectively captured by concepts such as ‘altmetrics’ [5]. We contend, however, that the most common altmetrics are not measuring impact, insofar as impact relates to the effect of research on clinical practice or thinking. Moreover, the definitions of various terms are not clear and they mean different things to different people. For the purposes of this experiment, we define the key concepts as set out in Figure 1. We took ‘reach’ to be the number of people who have been alerted to the presence of a web page and have the opportunity to view it [6]. Reach reflects the number of people who could potentially see the blog, either directly because they subscribe to the blog through RSS feed or email alerts, or through following the blog on various social media sites for example Facebook, Twitter, LinkedIn, Google+ or ResearchBlogging. One step closer to impact is engagement, defined here as the number of people who view the web page and then do something in response to viewing it – for example they ‘like’ it, re-tweet it, or they share it with their friends. The concept of ‘virality’ attempts to capture a stronger level of engagement and a reflection of the propensity of the message to ‘go viral’. Here we use the percentage of engagers who then write a story on the post on Facebook or begin a new tweet. This distinction between terms is important because as few as 16% of Facebook followers actually read a new post and about 1% of people who see and ‘like’ a Facebook page actually comment on it or start a new story on it [7]–[8].

A substantial gap in our understanding of the link between social media and impact, is the effect that a social medial release about a research article has on dissemination of the article itself. Remarkably, despite the apparent acceptance of social media reach, engagement and virality being evidence of impact, there seems to be no empirical evidence to support this claim [9]–[10]. This observation was recently noted by Priem et al - ‘Researchers must ask if altmetrics really reflect impact, or just empty buzz’ [5]. We undertook a blinded, randomised repeated measures experiment to test the hypothesis that social media release of an original research article in the clinical pain sciences increases viewing and downloads of the article, thereby demonstrating increased dissemination of the research and end-user behavioural change.

## Methods

Sixteen original research articles were selected from the PLOS ONE group of journals (Table S2). Inclusion criteria were: (i) relevance to the clinical pain sciences; (ii) of interest to the readership of our research group’s blog (bodyinmind.org), a readership that consists primarily of clinicians who work in a pain-related field; (iii) first published on-line between 01/01/2006 and 31/12/2011; (iv) not previously mentioned in a bodyinmind.org blog post.

**Figure 2:**
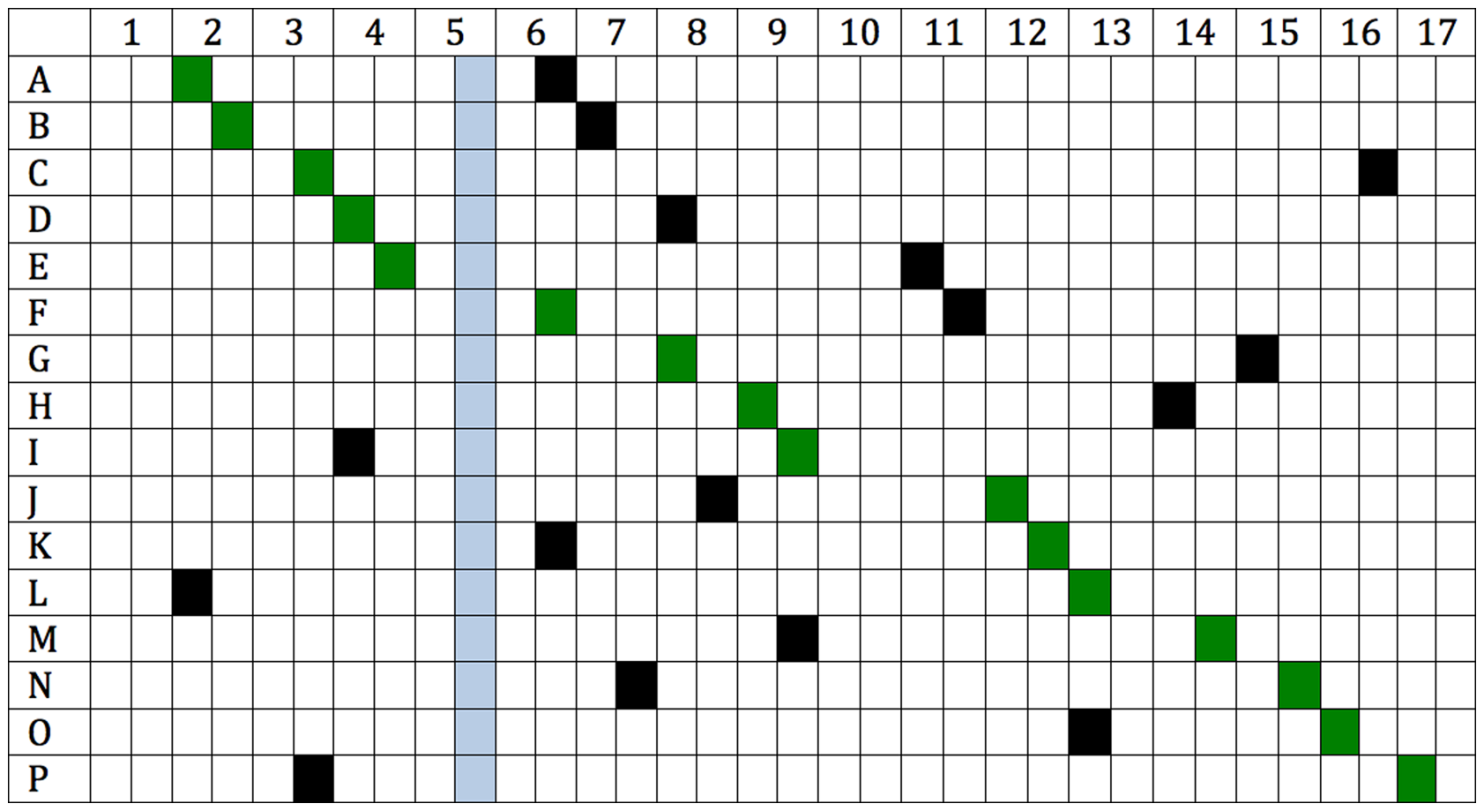
Social media release of articles. Days of the social media release for each article (A–P) are shown by green cells. The randomly selected control days are shown by black cells. The period during which PLOS citation tracking was down and therefore data are missing, is shown by blue cells.

Research articles were randomly allocated to four researchers in our group, each of whom wrote a blog post of around 500 words based on the original article, and which included a tag line directing the reader to the on-line version of the article for more information. All posts were released on a Tuesday (between 6 and 7 am) or between 11 pm Thursday and 2 am Friday, Australian Eastern Summer time. Other posts, not part of the current experiment, were also released during the experimental period (14/08/2011–02/02/2012). For each blog on a research article, two dates were randomly selected from all possible post-dates during the experimental period. Of the two dates, one was randomly selected as the release date and one as the control date (Fig. 2). Each blog post was broadcast via ResearchBlogging.org, Facebook, Twitter and LinkedIn on the day of the blog post. The experiment was undertaken covertly, so there was no risk that end-users who knew the experiment was being conducted would visit the original article as a result of that knowledge.

The primary outcome variables were rate of HTML views and PDF downloads over a seven day period. The former reflects some engagement with the target article by visiting it on the PLOS website. The latter reflects a higher level of engagement with the target article by adding it to a user library, presumably for future reference. Both outcome variables represent a behavioural change associated with the target article. Each primary outcome variable was assessed during four seven-day periods: the seven days before and after the release date, and the seven days before and after the control date.

Facebook statistics were provided by ‘Facebook page insights’ for 28 days post publication of the blog post. They are not broken down into individual days. Twitter comments and re-tweets were searched for manually and relied on the Twitter search engine to identify all mentions.

### Statistical Analysis

We undertook a 2×2 repeated-measures ANOVA on each primary outcome variable. The first factor was ‘Date’ (two levels: release date or control date). The second factor was ‘Week’ (two levels: before date or after date). In order to maximise the likelihood of detecting an effect on each primary outcome variable, which we took to reflect different levels of dissemination, we did not correct for multiple measures and set α = 0.05.

### Secondary Analyses - Relating Citations, HTML Views and PDF Downloads to Social Media Reach and Engagement

We calculated the relationship between the primary outcome variables and recognised measures of social media reach and social media engagement. We undertook two linear regressions with the increase in HTML views or PDF downloads as the dependent variable, and the following measures of social media reach and engagement as the independent variables:

#### Reach

The number of unique visitors who were alerted to the blog post and had the opportunity to view it.

#### Engagement

The number of unique people who liked, commented on, or shared the blog post on www.bodyinmind.org, Facebook, Twitter or LinkedIn.

#### Virality

The percentage of unique viewers who then created a story from the blog post on Facebook, twitter, or blogged about it separately.

We investigated whether social media reach or engagement related to a conventional measure of impact - citation count, as provided by Scopus. We did this using a third linear regression, with reach and engagement as regressors, and citation count at 03/09/2012 as the dependent variable. We also investigated whether HTML views or PDF downloads related to citations by correlating citation count at 03/09/2012 with total HTML views and total PDF downloads at the end of the week after the social medial release.

We tested whether there was a ‘blogger effect’ (ie, do some blogger’s posts have a greater impact than others?) by first calculating the difference in the change or rate of increase in the primary outcome variables between the social media release date and the control date. We called this the blog effect. We then compared the blog effect between reviewers using a Kruskal-Wallis test. Finally, we tested whether there was an ‘age effect’ (ie, is there an effect of the age of the article on our outcome variables?) by relating the blog effect to the days between publication of the article and the social media release.

No correction was applied for multiple measures because these were secondary and therefore exploratory, hypothesis-generating analyses.

## Results

Over the 18-week study period, the blog (bodyinmind.org) had an average of 2585 unique views per week. Each post was viewed a mean (SD) of 507 (160) times in the week following publication. In the 28 days after publication, a mean (SD) of 693 (135) unique visitors saw the post in their Facebook newsfeed; 35 (16) unique visitors clicked on each post; 6 (4) unique visitors created a like, comment or share from the post. Of the total number of unique visitors who saw the post on Facebook, 0.93% (0. 66%) created a story from it.

### HTML Views

**Figure 3:**
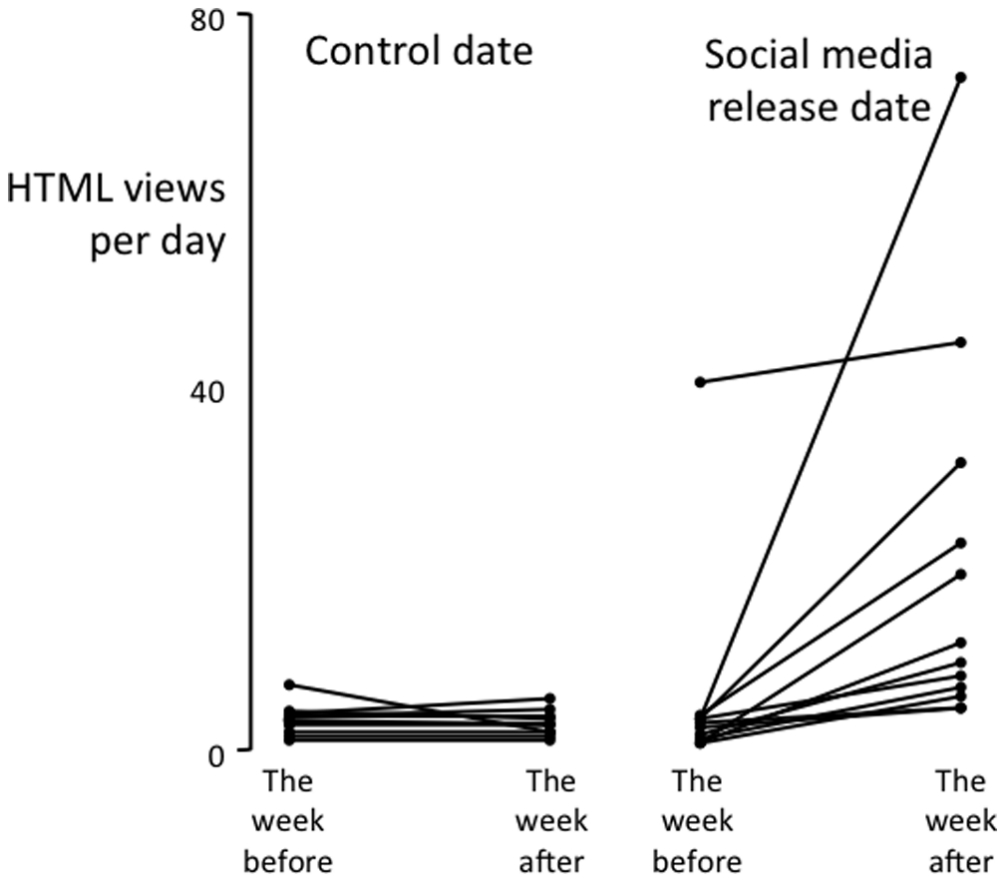
The effect of social medial release on HTML views of the original article. The rate of HTML views of each research article on which a social media release was based, for the week either side of two randomly selected dates. The data for the control date are on the left and the data for the social medial release date are on the right. Note the systematic increase in rate of HTML views from the week before the social media release to the week after it.

The rate of HTML views was higher during the second week than during the first, regardless of the date. That is, there was a main effect of Week on HTML views (F(1,15) = 6.27, p = 0.024). The rate of HTML views was also higher either side of the social media release than it was either side of the control date (main effect of Date on HTML views – F(1,15) = 7.39, p = 0.016). However, visual inspection of the data (Fig. 3) show that these main effects were driven to a large extent by an interaction, such that the social media release was associated with a larger increase in the rate of HTML views than the control date was (Week x Date interaction: F(1,15) = 7.39, p 0.016). The mean ± SD rate of HTML views in the week after the social media release was 18±18 per day, whereas the rate during the other three weeks was no more than 6±3 per day (Fig. 3), which equates to an effect size (Cohen’s d) of 0.9. That is, in the week after the social media release, about 12 people per day viewed the research article as a result of the social medial release.

### PDF Downloads

**Figure 4:**
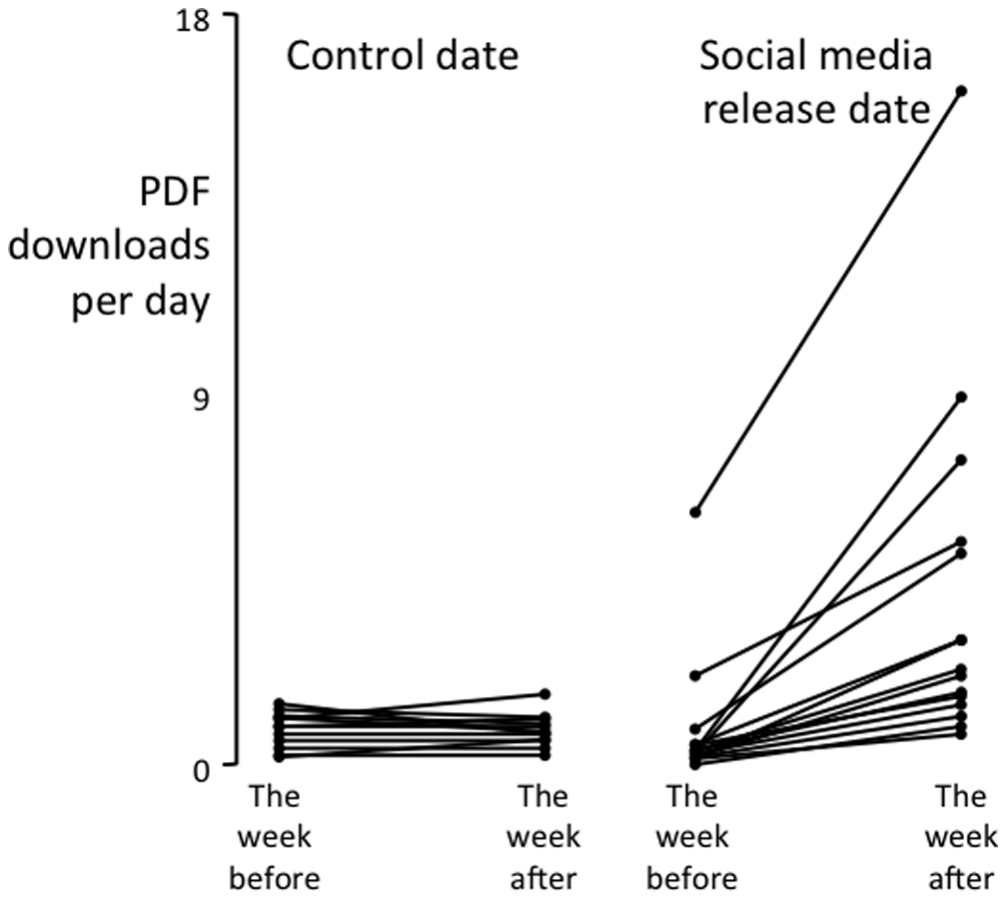
The effect of social medial release on PDF downloads of the original article. The rate of PDF downloads of each research article on which a social media release was based, for the week either side of two randomly selected dates. The data for the control date are on the left and the data for the social medial release date are on the right. Note the systematic increase in rate of PDF downloads from the week before the social media release to the week after it.

The results for PDF downloads reflected those for HTML views: The rate of PDF downloads was higher during the second week than during the first (main effect of Week – F(1,15) = 10.83, p = 0.005) and higher either side of the social media release than it was either side of the control Date (main effect of date – F(1,15) = 6.57, p = 0.022). Again, these effects were driven by an interaction, such that the social media release was associated with a larger increase in the rate of PDF downloads than the control date was (Week x Date interaction: F(1,15) = 14.74, p = 0.002). The mean ± SD rate of PDF downloads in the week after the social media release was 4±4 per day, whereas the rate during the other three weeks was less than 1±1 per day (Fig. 4), which equates to an effect size (Cohen’s d) of 1. That is about 3 people per day downloaded the research article as a result of the social media release.

### How well do reach, engagement and virality of the social media release relate to HTML views and PDF downloads of the research article?

Engagement was 5.3% of reach and virality was 0.9% of engagement. None of the social media metrics related to the increase in rate of HTML views of the research article (p = 0.947 for reach; p = 0.809 for engagement; p = 0.544 for virality), nor to the increase in PDF downloads of the research article (p = 0.323 for reach; p = 0.864 for engagement; p = 0.934 for virality). The only relationship that approached significance was that between the number of HTML views of the blog post and PDF downloads (p = 0.09).

### Relationship between Reach, Virality and Citations

There was no relationship between citations on Scopus about one year after publication and any of the social media metrics (p>0.68 for all). Total PDF downloads at the end of the week after social media release related to total HTML views at the same time (Pearson r = 0.72; p = 0.002). Interestingly, citations at 03/09/2012 related to total PDF downloads (Pearson r = 0.51; p = 0.045) but not to total HTML views (Pearson r = 0.06; p = 0.826).

### Was there a ‘blogger’ Effect?

One blogger wrote seven posts, two wrote four posts and one wrote one post. There was no difference between bloggers for either HTML views or PDF downloads (p>0.88 for both).

### Was there an Article Age Effect?

The age of the article at the time of blogging was not related to the rate of HTML views, or the rate of PDF downloads, during the any of the four one-week periods (p>0.71 for all). The blog effect was not affected by the age of the article at the time of blogging (p = 0.28).

## Discussion

We hypothesised that social media release of an original research article in the clinical pain sciences increases viewing and downloads of the article. The results support our hypothesis. In the week after the social media release, there were about 12 extra views of the HTML of the research article per day, and 3 extra downloads of the article itself per day, that we can attribute to the social media release. The effects were variable between articles, showing that multiple factors mediate the effect of a social media release on our chosen outcome variables. Although the absolute magnitude of the effect might be considered small (about 0.01% of people we reached were sufficiently interested to download the PDF), the effect size of the intervention was large (Cohen’s d >0.9 for both outcomes). The effect of social media release was probably smaller for our site, which is small, young and specialised, than it would be for sites with greater gravitas, for example NEJM or BMJ or indeed, PLOS.

### Relationship between Reach and Impact

The idea of social media reach is fairly straightforward - it can be considered as the number of people in a network, for example the number of Facebook friends or Twitter followers. A blog may have 2,000 Facebook ‘likes’, 700 Twitter followers and 300 subscribers - a reach of three thousand people. Impact is less straightforward. As depicted in Figure 1, the various definitions of social media each reflects a substantially larger population than our most proximal measure of impact – HTML views and PDF downloads of the original article. One might suggest that impact should reflect some sense of engagement with the material, for example the number of people within a network who make a comment on a post. From a clinical pain sciences perspective, change in clinical practice or clinician knowledge would be clear signs of impact, but such metrics are very difficult to obtain. Perhaps this is part of the reason that researchers are using, we believe erroneously, social media reach as a measure of social media impact.

There are now several social media options that researchers integrate into their overall ‘impact strategy’, for example listing their research on open non-subscription sites such as Mendeley, and joining discussions about research on social media sites such as Twitter and on blogs. Certainly, current measures of dissemination, most notably citations of articles or the impact factor of the journals in which they are published, do not take into account the social media impact of the article. New measurements, such as altmetrics [5] and article-level metrics such as those provided by PLOS [11], aim to take into account the views, citations, social network conversations, blog posts and media coverage in an attempt to analyse the influence of research across a global community. There is merit in this pursuit, but, although our study relates to clinical pain sciences research, our results strongly suggest that we need to be careful in equating such measures with impact or influence, or using them as a surrogate for dissemination. Indeed, not even virality, which estimates the propensity of an item to ‘go viral’, was related with HTML views or PDF downloads. This is very important because our results actually suggest that we may be measuring the wrong thing when it comes to determining the social media impact of research. That is, we showed a very clear effect of the social media release on both HTML views and PDF downloads of the target article. However, we did not detect any relationship between either outcome and the social media metrics we used. The only variable that related to either outcome was the number of HTML views, of the original blog post, in the week after social media release. It seems clear then, that it is not the total number of people you tell about your study, nor the number of people they tell, nor the number of people who follow you or who re-tweet your tweets. In fact, it appears that we are missing more of how the release improves dissemination than we are capturing.

The final result, that citation count did not relate to any social media measures, casts doubt over the intuitively sensible idea that social media impact reflects future citation-related impact [12]. We used the Scopus citation count, taken almost 9 months after the completion of the experimental period, and 1–2 years after the publication date of the target articles, as a conventional measure of impact. There was no relationship between citation count and our measures of social media reach or virality. One must be cautious when interpreting this result because citation count so soon (1–2 years) after publication might be unlikely to capture new research that was triggered by the original article – although, importantly, journal impact factors are calculated on the basis of citations in the two years after publication. Suffice here to observe that the apparent popularity of an article on social media does not necessarily predict its short-term citation count.

Although this is the first empirical evaluation of social media impact in the clinical pain sciences and we have employed a conservative and robust design, we acknowledge several limitations. Social media dissemination in the clinical sciences relies on clinicians having access to, and using, social media. It will have no effect for those who do not use the web and who rely on more traditional means of dissemination - ‘pulling’ the evidence. Although there was an increase in HTML views and PDF downloads as a result of social media dissemination, we do not know if people read the article or whether it changed their practice. We presumed that a portion of those who viewed the HTML version of the article would then go onto download it, however our data suggest that a different pattern of access is occurring. Unfortunately, our data do not allow us to determine whether the same people both viewed the HTML and downloaded the article PDF or whether different people viewed the HTML and downloaded the article PDF. Downloading a PDF version of a paper does not necessarily imply that they would later read it, but it does increase the probability of such.

Citations and impact factors measure the impact within the scientific community whereas views by social media will also include interested clinicians and laypeople and, as such, measure uptake by different audiences. Although we used a variety of different social media platforms to disseminate to as wide an audience as possible, we do not know who the audience is - we can only surmise that they are a mixture of researchers, clinicians, people in pain and interested laypeople. Further, each social media strategy comes with inherent limitations in regards to data collection of usage statistics related to a blog post. Gathering Facebook and Twitter statistics for each article is still cumbersome and is probably not always accurate. The risk in using search engines to gather data is that there is no way of knowing whether all the data have been identified. For Twitter there is no way to retrospectively calculate the number of re-tweets accurately over a longer period retrospectively for each post [13]. As a result, our Twitter data is a best estimate and my have underestimated the true values but, critically, we would expect this effect to be unrelated to our blog post and therefore not impact on our findings. Regarding Facebook, shares, likes and comments are grouped as one statistic but in reality only shares and comments show engagement with the post and indicate that people are more likely to have read it. Regarding LinkedIn, the only available data was the number of members of the BodyInMind group and as such, we have no way of knowing how many viewed the actual blog post.

The blog, BodyInMind.org, through which the original blog posts of PLoS ONE articles were released, experienced a technical interruption half-way through the experiment. In spite of an attempt by PLOS to retrieve the statistics, approximately five days of data were lost on several of the blog posts. This also meant that additional data on traffic, such as percentage of traffic for each blog post from external sources such as Facebook, Twitter, LinkedIn and ResearchBlogging could not be measured during this period. Critically and fortuitously, this period did not coincide with data collection weeks (see Fig. 2). PLOS indicated that this technical problem has now been fixed, but similar problems may arise in the future and present an ongoing risk to studies such as ours. Although disconcerting for those keenly following social media data, this problem would be very unlikely to have affected our primary outcomes because none of our dates fell within the period that was affected.

Social influence can produce an effect whereby something that is popular becomes more popular and something that is unpopular becomes even less popular [14]. It seems possible that articles on BodyInMind.org were shared because the site is popular among a discrete community and not because the article itself merited circulation. This possibility does not confound our main result but it adds a possible argument to the common objective of making a blog more popular as a device to boost social media impact of individual posts. Finally, our study relied on the target articles being freely available to the public. Many journals are not open access, particularly those in the clinical pain sciences. Therefore, we must be cautious extrapolating our results to subscription only access journals.

In conclusion, our results clearly support the hypothesis that social media can increase the number of people who view or download an original research article in the clinical pain sciences. However, the size of the effect is not related to conventional social media metrics, such as reach, engagement and virality. Our results highlight the difference between social media reach and social media impact and suggest that the latter is not a simple function of the former.

## Supporting Information

Table S1 **Example of Journals social media use as viewed 9 August 2012.**
(XLS)

Table S2 **PLoS articles dissemination and corresponding changes in views and downloads.** (*)Reach = The number of unique people who have seen the post in their newsfeed or on the Body in Mind page. Figures are for the first 28 days after a posts’s publication only. None of the posts were promoted via Facebook advertisements. (†)Engaged Users = number of unique people who have clicked on your post. Figures are for the first 28 after a post’s publication only. (‡)Talking about this = the number of unique people who have created a ‘story’ (a like, comment on, or share) from the post. Figures are for the first 28 days after publication only. (§)Virality = the percentage of people who have created a story from the post out of the total number of unique people who have seen it. (**)Number of tweets as of 28 March 2012.
(DOC)
