# Supplementary material for: Social Media Release Increases Dissemination of Original Articles in the Clinical Pain Sciences
Source: PLoS One. 2013 Jul 17;8(7):e68914. doi: 10.1371/journal.pone.0068914 (PMC3714259; doi:10.1371/journal.pone.0068914)
Supplement: Table S2 — PLoS articles dissemination and corresponding changes in views and downloads. (*)Reach = The number of unique people who have seen the post in their newsfeed or on the Body in Mind page. Figures are for the first 28 days after a posts’s publication only. None of the posts were promoted via Facebook advertisements. (†)Engaged Users = number of unique people who have clicked on your post. Figures are for the first 28 after a post’s publication only. (‡)Talking about this = the number of unique people who have created a ‘story’ (a like, comment on, or share) from the post. Figures are for the first 28 days after publication only. (§)Virality = the percentage of people who have created a story from the post out of the total number of unique people who have seen it. (**)Number of tweets as of 28 March 2012. (DOC) [file pone.0068914.s002.doc]

Table S2. PLoS articles dissemination and corresponding changes in views and downloads

| Blog post nr | PLoS article | Nr of blog views 1 week post publication | Facebook | | | | Nr of Tweets (**) | Change in HTML views 7 days post publication | Change in PDF downloads 7 days post publication |
| --- | --- | --- | --- | --- | --- | --- | --- | --- | --- |
|  |  |  | Reach (*) | Engaged users (†) | Talking about this (‡) | Virality (%) (§) |  |  |  |
| 3 | Andersen JH, Fallentin N, Thomsen JF, Mikkelsen S (2011) Risk Factors for Neck and Upper Extremity Disorders among Computers Users and the Effect of Interventions: An Overview of Systematic Reviews. PLoS ONE 6(5): e19691. doi:10.1371/journal.pone.0019691 | 465 | 980 | 45 | 8 | 0.82 | 9 | 58 | 17 |
| 2 | "Hägni K, Eng K, Hepp-Reymond M-C, Holper L, Keisker B, et al. (2008) Observing Virtual Arms that You Imagine Are Yours Increases the Galvanic Skin Response to an Unexpected Threat. PLoS ONE 3(8): e3082. doi:10.1371/journal.pone.0003082 | 408 | 567 | 39 | 9 | 1.59 | 10 | 25 | 6 |
| 1 | Huge V, Lauchart M, Magerl W, Beyer A, Moehnle P, et al. (2011) Complex Interaction of Sensory and Motor Signs and Symptoms in Chronic CRPS. PLoS ONE 6(4): e18775. doi:10.1371/journal.pone.0018775 | 643 | 548 | 63 | 13 | 2.23 | 18 | 221 | 57 |
| 6 | "Marzoli D, Palumbo R, Di Domenico A, Penolazzi B, Garganese P, et al. (2011) The Relation between Self-Reported Empathy and Motor Identification with Imagined Agents. PLoS ONE 6(1): e14595. doi:10.1371/journal.pone.0014595 | 392 | 747 | 15 | 1 | 0.13 | 1 | 69 | 12 |
| 5 | Mazzola V, Latorre V, Petito A, Gentili N, Fazio L, et al. (2010) Affective Response to a Loved One's Pain: Insula Activity as a Function of Individual Differences. PLoS ONE 5(12): e15268. doi:10.1371/journal.pone.0015268 | 422 | 765 | 20 | 1 | 0.13 | 19 | 569 | 12 |
| 4 | Petkova VI, Ehrsson HH (2009) When Right Feels Left: Referral of Touch and Ownership between the Hands. PLoS ONE 4(9): e6933. doi:10.1371/journal.pone.0006933 | 589 | 597 | 51 | 8 | 1.34 | 18 | 5 | 3 |
| 7 | Elliott J, Pedler A, Kenardy J, Galloway G, Jull G, et al. (2011) The Temporal Development of Fatty Infiltrates in the Neck Muscles Following Whiplash Injury: An Association with Pain and Posttraumatic Stress. PLoS ONE 6(6): e21194. doi:10.1371/journal.pone.0021194 | 635 | 668 | 42 | 4 | 0.6 | 20 | 158 | 47 |
| 10 | Leknes S, Lee M, Berna C, Andersson J, Tracey I (2011) Relief as a Reward: Hedonic and Neural Responses to Safety from Pain. PLoS ONE 6(4): e17870. doi:10.1371/journal.pone.0017870 | 683 | 550 | 54 | 9 | 1.64 | 18 | 52 | 4 |
| 8 | Mehling WE, Gopisetty V, Daubenmier J, Price CJ, Hecht FM, et al. (2009) Body Awareness: Construct and Self-Report Measures. PLoS ONE 4(5): e5614. doi:10.1371/journal.pone.0005614 | 935 | 640 | 57 | 12 | 1.88 | 26 | 19 | 68 |
| 9 | Sanchez-Vives MV, Spanlang B, Frisoli A, Bergamasco M, Slater M (2010) Virtual Hand Illusion Induced by Visuomotor Correlations. PLoS ONE 5(4): e10381. doi:10.1371/journal.pone.0010381 | 299 | 536 | 23 | 3 | 0.56 | 14 | 84 | 3 |
| 15 | Canessa N, Motterlini M, Di Dio C, Perani D, Scifo P, et al. (2009) Understanding Others' Regret: A fMRI Study. PLoS ONE 4(10): e7402. doi:10.1371/journal.pone.0007402 | 426 | 805 | 15 | 1 | 0.12 | 7 | 8 | 9 |
| 13 | Lamm C, Nusbaum HC, Meltzoff AN, Decety J (2007) What Are You Feeling? Using Functional Magnetic Resonance Imaging to Assess the Modulation of Sensory and Affective Responses during Empathy for Pain. PLoS ONE 2(12): e1292. doi:10.1371/journal.pone.0001292 | 377 | 575 | 23 | 6 | 1.04 | 11 | 37 | 6 |
| 12 | Pujol J, López-Solà M, Ortiz H, Vilanova JC, Harrison BJ, et al. (2009) Mapping Brain Response to Pain in Fibromyalgia Patients Using Temporal Analysis of fMRI. PLoS ONE 4(4): e5224. doi:10.1371/journal.pone.0005224 | 445 | 832 | 24 | 3 | 0.36 | 22 | 54 | 19 |
| 11 | Krach S, Cohrs JC, de Echeverría Loebell NC, Kircher T, Sommer J, et al. (2011) Your Flaws Are My Pain: Linking Empathy To Vicarious Embarrassment. PLoS ONE 6(4): e18675. doi:10.1371/journal.pone.0018675 | 488 | 850 | 29 | 4 | 0.47 | 8 | 41 | 2 |
| 14 | Lass-Hennemann J, Kuehl LK, Schulz A, Oitzl MS, Schachinger H (2011) Stress Strengthens Memory of First Impressions of Others' Positive Personality Traits. PLoS ONE 6(1): e16389. doi:10.1371/journal.pone.0016389 | 556 | 811 | 42 | 9 | 1.11 | 62 | 26 | 7 |
| 16 | Thakkar KN, Nichols HS, McIntosh LG, Park S (2011) Disturbances in Body Ownership in Schizophrenia: Evidence from the Rubber Hand Illusion and Case Study of a Spontaneous Out-of-Body Experience. PLoS ONE 6(10): e27089. doi:10.1371/journal.pone.002708 | 346 | 611 | 21 | 5 | 0.82 | 40 | 36 | 22 |

(*)Reach = The number of unique people who have seen the post in their newsfeed or on the Body in Mind page. Figures are for the first 28 days after a posts’s publication only.

None of the posts were promoted via Facebook advertisements

(†)Engaged Users = number of unique people who have clicked on your post. Figures are for the first 28 after a post’s publication only.

(‡)Talking about this = the number of unique people who have created a ‘story’ (a like, comment on, or share) from the post. Figures are for the first 28 days after publication only.

(§)Virality = the percentage of people who have created a story from the post out of the total number of unique people who have seen it.

(**)Number of tweets as of 28 March 2012
